# Supplementary material for: The epidemic characteristics of Mycoplasma pneumoniae infection among children in Anhui, China, 2015–2023
Source: Microbiol Spectr. 2024 Sep 3;12(10):e00651-24. doi: 10.1128/spectrum.00651-24 (PMC11448379; doi:10.1128/spectrum.00651-24)
Supplement: Supplemental figures — Fig. S1 and S2. [file spectrum.00651-24-s0001.docx]

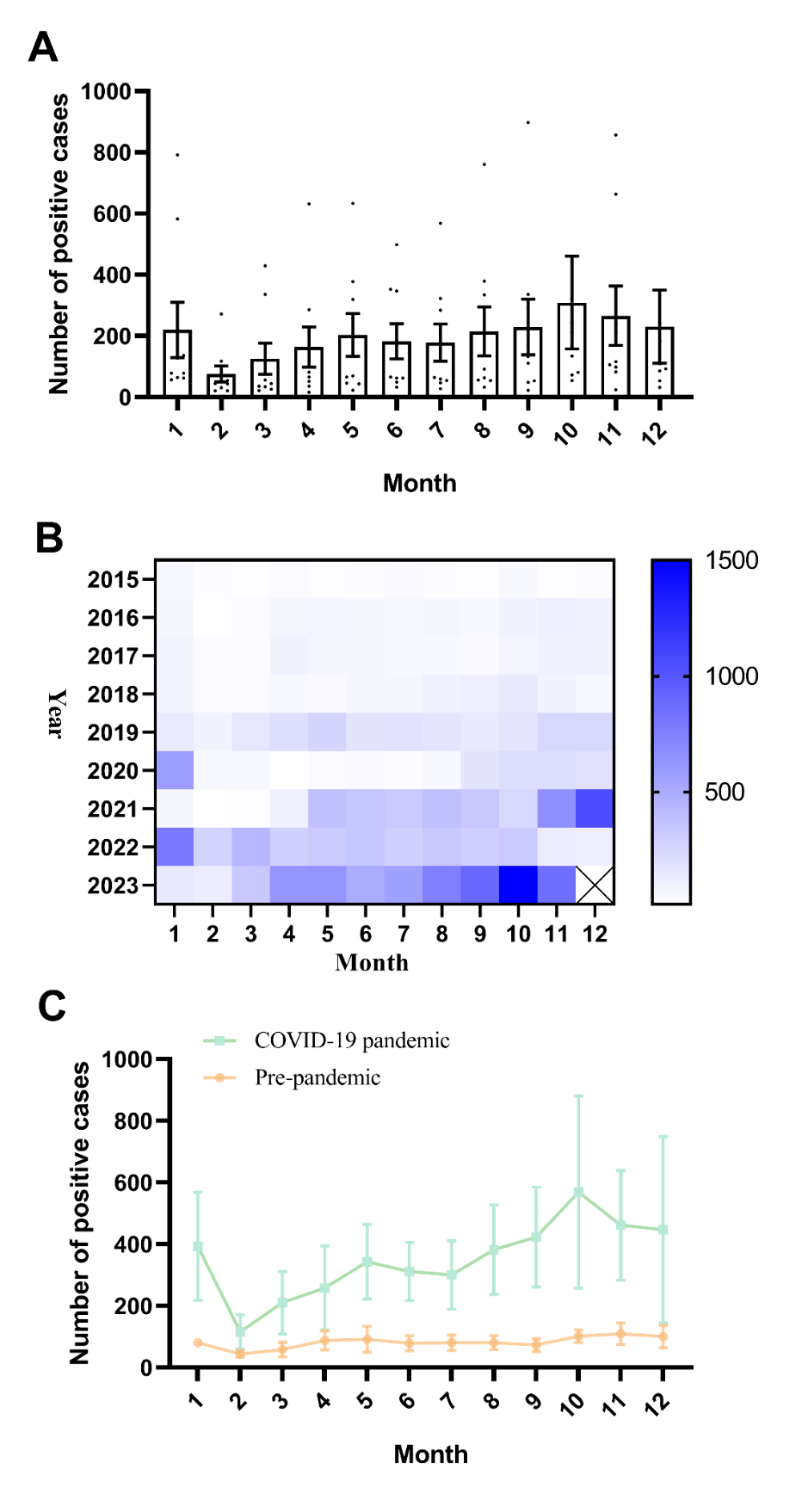


Supplementary Figure S1. Changes in the number of *M. pneumoniae* positive cases in different months. (A) Changes in the number of positive cases of *M. pneumoniae* in different months. (B) The changes in the number of positive cases of *M. pneumoniae* in different months from 2015 to 2023 were demonstrated by heatmap. (C) Changes in the number of positive cases of *M. pneumoniae* in different months before and after the COVID-19 outbreak.


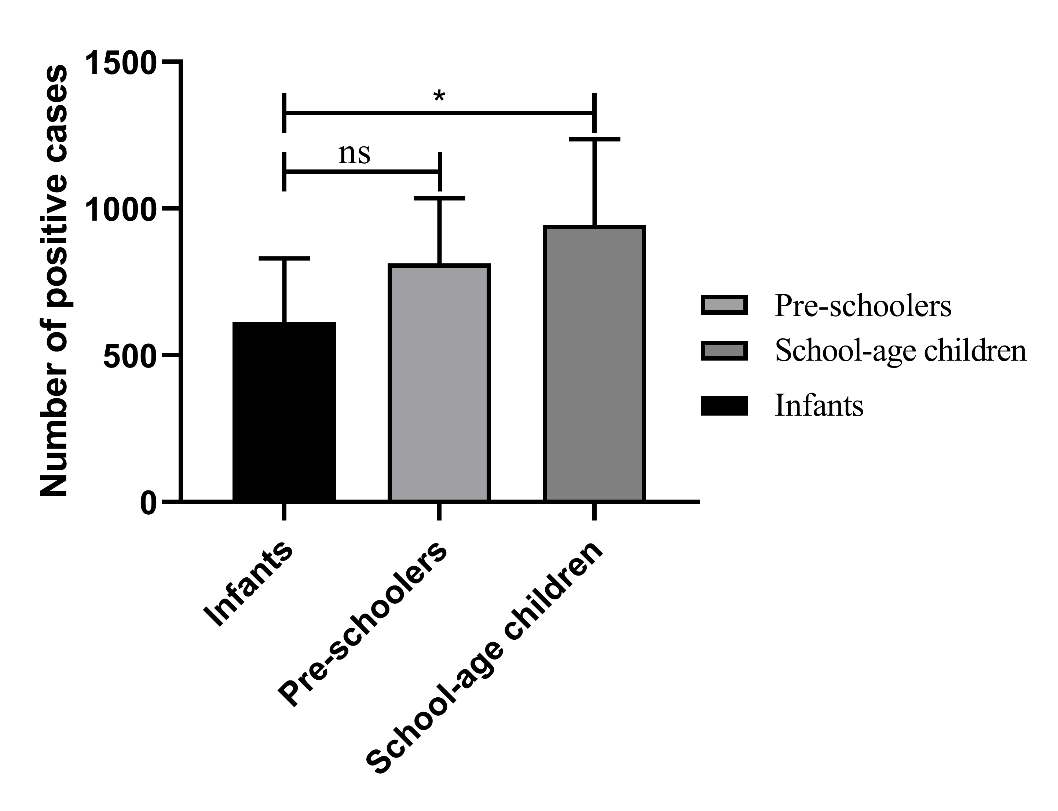


Supplementary Figure S2. Difference of the number of positive cases of *M. pneumoniae* in different age groups, ns: No significance, *:P<0.05.
